# A *CACNA2D2*‐Related Recessive Form of Cerebellar Abiotrophy in Angus Cattle

**DOI:** 10.1002/age.70086

**Published:** 2026-03-24

**Authors:** Joana Jacinto, Francesca Chianini, Jo Moore, Timothy Geraghty, Irene M. Häfliger, Franz R. Seefried, Alwyn Jones, Helen Carty, Anna Letko, Cord Drögemüller

**Affiliations:** ^1^ Clinic for Ruminants, Vetsuisse Faculty University of Bern Bern Switzerland; ^2^ Institute of Genetics, Vetsuisse Faculty University of Bern Bern Switzerland; ^3^ The Moredun Research Institute Penicuik UK; ^4^ Labcorp Harrogate UK; ^5^ SRUC Veterinary Services, Mill of Craibstone, Bucksburn Aberdeen UK; ^6^ Qualitas AG Zug Switzerland

**Keywords:** brain, cattle, large animal model, precision medicine, WGS

## Abstract

Cerebellar disease in ruminants is often virus‐induced and non‐genetic, but there are also rare inherited forms of cerebellar hypoplasia and cerebellar abiotrophy (CA). So far, no causal variant has been reported for these conditions in cattle. Two inbred Angus calves suspected of having cerebellar disease were reported in Scotland. The aims of this study were to characterize the clinicopathological phenotype of Angus calves affected by a cerebellar disease, to identify a causal variant assuming autosomal monogenic recessive inheritance and to evaluate its prevalence in Angus populations. Clinicopathological investigations were performed, including the exclusion of prevalent teratogenic viruses as well as a multiple‐case whole‐genome sequencing (WGS) approach. The two affected Angus calves showed congenital intention tremor and brain examination detected cerebellar abiotrophy. Genetic analysis identified a private homozygous missense variant in the bovine *CACNA2D2* gene (XP_024839037.1:p.(Cys395Arg)), which is linked to neurological disorders in other species, including a form of cerebellar atrophy in humans. This variant was classified as pathogenic and shown to be absent in sequence data from over 5000 other cattle with available WGS data as well as in a cohort of 16 purebred Angus cattle from Switzerland. The variant is proposed to cause a rare form of CA in Angus and therefore should be monitored in the Angus global population, as previous similar cases were reported elsewhere. For the first time, we characterized a genetic form of cerebellar disease in cattle, providing the first large animal model for a condition related to the *CACNA2D2* gene.

## 1 Introduction

Cerebellar disease in young ruminants may be present at birth or develop later, with clinical signs that vary according to the underlying pathology. In cattle, two well‐recognized cerebellar disorders are cerebellar hypoplasia and cerebellar abiotrophy (CA). Cerebellar hypoplasia is a congenital condition in which the cerebellum is underdeveloped and reduced in size, resulting in non‐progressive signs from birth. In contrast, CA is a degenerative disorder in which the cerebellum initially forms normally, but Purkinje cells within the cerebellar cortex undergo progressive loss, leading to a gradual onset and worsening of clinical signs (Gibbons 2017).

Cerebellar hypoplasia in cattle can be accompanied by cerebral abnormalities and represents one of the most common congenital malformations associated with intrauterine viral infections (Agerholm et al. 2015; Gallina et al. 2021). However, rare inherited forms have also been reported in other domestic animal species such as dogs (OMIA:001947‐9615) (Gerber et al. 2015), yet no causal variant has been identified in cattle (OMIA:000179‐9913).

Cerebellar abiotrophy is most often heritable following autosomal monogenic inheritance, and offspring are typically born normal and develop neurologic signs later in life (Gibbons 2017). Representing a neurodegenerative disorder, CA, also known as neonatal cerebellar cortical degeneration, is clinically characterized by progressive cerebellar dysfunction, most notably ataxia, hypermetria, a wide‐based stance, and tremors (Scott et al. 2018). Histologically, CA is characterized by the selective degeneration and loss of Purkinje cells in the cerebellar cortex (Scott et al. 2018). This is accompanied by secondary loss of granule cells, swollen Purkinje axons, astrogliosis, and occasional white matter changes such as Wallerian degeneration (Scott et al. 2018). Definitive confirmation can be achieved through post‐mortem histopathology, which shows loss of Purkinje cells.

Several breed‐specific, recessively inherited forms of CA in domestic animals such as dogs (OMIA:002602‐9615) and horses (OMIA:000175‐9796) have been shown to be caused by variants affecting different single genes (Scott et al. 2018). After the first description of CA in Ayrshire cattle in 1951 (Jennings and Sumner 1951), the disorder has been observed in several other breeds of cattle, such as Angus, Hereford, and Holstein (OMIA:000175‐9913) (Jennings and Sumner 1951; Kemp et al. 1995; Mitchell et al. 1993; Schild et al. 2001; Whittington et al. 1989), but so far no molecular characterization has been done. Therefore, as is already known for dogs, genetic heterogeneity for bovine CA within and across breeds is also suspected as different forms of CA were reported with variable age of onset, from a few days to 2 years of age (Mitchell et al. 1993; Whittington et al. 1989). For example, the later onset of cerebellar signs suggests that Australian cases of CA in Angus cattle differ from the “familial convulsions and ataxia” described in Angus cattle in Scotland and New Zealand (Windsor et al. 2011).

This study aimed to (1) characterize the clinicopathological findings of Angus calves affected by CA; (2) investigate the potential genetic basis of the condition in two calves born from consanguineous matings, under the hypothesis of a recessive, single‐gene disorder; and (3) evaluate the allele frequency of the implicated variant across different Angus populations.

## 2 Materials and Methods

### 2.1 Animals, Clinicopathological Investigation and Exclusion of Prevalent Teratogenic Viral Infections

Two purebred Angus calves showing neurological signs from birth were reported to the SRUC and Moredun Research Institute in 2020. The two Angus cases originated from the same herd and reportedly resulted from accidental inbreeding.

Case 1 was a heifer calf born naturally. Case 2 was a bull calf also born naturally. According to the owner, the calf showed abnormal behavior immediately after birth, including marked incoordination, persistent head nodding, and repeated neck extension. The calf was unable to rise and required supplementary feeding including delivery of colostrum via stomach tube followed by handfeeding for 4 days. During this time, the calf could suckle when assisted but was never able to stand up or remain standing on its own. Case 2 was a bull calf also born naturally. The calf presented with the same neonatal abnormalities as case 1, with severe neurological deficits evident at birth, slight transient improvement, and persistent inability to stand unassisted.

The cases underwent a thorough clinical examination on farm and were subsequently euthanized due to the poor prognosis and submitted for post‐mortem examination. Case 1 was 4 days old at examination and case 2 was 1 day old.

Brain and spinal cord were fixed in 10% neutral buffered formalin. Once completely fixed, tissues were trimmed, dehydrated and embedded in paraffin. Five micrometer sections were cut from the paraffin blocks and used to prepare histological glass slides which were stained with hematoxylin and eosin (H&E).

To investigate potential viral etiologies, brain samples from both calves were tested for the presence of bovine viral diarrhea virus (BVDV), bluetongue virus (BTV), and Schmallenberg virus (SBV) using PCR.

### 2.2 DNA Extraction

Genomic DNA was extracted from ear tissue samples from both calves using Promega Maxwell RSC DNA system (Promega, Dübendorf, Switzerland).

### 2.3 Whole‐Genome Sequencing and Variant Filtering

WGS data were generated using the Illumina NovaSeq6000 platform (Illumina Inc., San Diego, CA, USA) from genomic DNA samples from the two calves. The sequenced reads were mapped to the ARS‐UCD1.2 reference genome (Rosen et al. 2020), resulting in an average read depth of 21×, and single‐nucleotide variants (SNV) and small indel variants were called. The applied software and steps to process fastq files into binary alignment map and genomic variant call format (VCF) files were in accordance with the 1000 Bull Genomes Project processing guidelines of run 7 (Hayes and Daetwyler 2019), except for the trimming, which was performed using fastp (Chen et al. 2018). Downstream processing of the genomic data was performed as reported previously (Häfliger et al. 2020). The effects of all called variants were functionally evaluated with snpEff v5.0c (Cingolani et al. 2012), using the NCBI Annotation Release 106. This resulted in the final VCF file and their functional annotations, comprising jointly genotyped individual variants of 1035 animals including the two cases, and 1033 diverse controls from the ongoing Swiss Comparative Bovine Resequencing project.

Considering the reported inbreeding loop, we performed variant filtering under the hypothesis of a recessive, breed‐specific monogenic defect. Accordingly, we assumed that the two affected individuals would be homozygous for the variant allele, and the controls would be either heterozygous or homozygous for the reference allele.

### 2.4 Runs of Homozygosity (ROH) and Genomic Inbreeding Analysis

PLINK v1.9 (Chang et al. 2015) was used for quality control pruning of the initial 49 965 295 variants called in 18 purebred Angus genomes extracted from the Swiss Comparative Bovine Resequencing project. Only high‐quality biallelic SNVs mapped to the 29 bovine autosomes called in all individuals with minor allele frequency > 0.05 and Hardy–Weinberg equilibrium exact test *p*‐value > 1 × 10^−6^ were retained for the downstream analyses, which constituted a set of 11 918 353 SNVs. Genome‐wide search for ROH in regions shared by the two calves was performed using the R package detectRUNS v.0.9.6 (Biscarini et al. 2019). Based on published guidelines (Gorssen et al. 2021), the following parameters were set for the ROH detection: sliding window size of minimum 20 SNVs, maximum of three heterozygous genotypes in a window, minimum ROH length of 200 kb, minimum number of 66 SNVs in an ROH, and minimum density of one SNV per 50 kb. Additionally, homozygosity‐based genomic inbreeding coefficient (F_ROH) was calculated based on the total length of the autosomal genome covered by SNV positions (herein 2 488 159 091 bp) and the F_ROH of the two studied cases were compared to the mean F_ROH obtained from the analysis of the 16 control Angus genomes.

### 2.5 Variant in Silico Assessment of Its Molecular Consequences

PredictSNP1 (Bendl et al. 2014), PolyPhen‐1, Polyphen‐2 (Adzhubei et al. 2013), SIFT (Ng and Henikoff 2003), PhD‐SNPg (Capriotti and Fariselli 2023), and MutPred2 (Pejaver et al. 2017) were used to predict the biological consequences of the only remaining candidate variant in *CACNA2D2*. Additionally, IGV software version 2.0 (Robinson et al. 2017) was used for visual inspection of the candidate variant resulting from variant filtering.

### 2.6 Occurrence of the CACNA2D2 Variant in a Global Control Cohort

The comprehensive variant catalog from run 9 of the 1000 Bull Genomes Project was available to investigate the allelic distribution of the identified candidate variant in *CACNA2D2* within a global control cohort (Hayes and Daetwyler, 2019). The full dataset comprised 5116 bovine genomes, including 576 from the Swiss Comparative Bovine Resequencing Project, from a wide variety of more than 130 breeds.

### 2.7 Targeted Genotyping of the CACNA2D2 Variant

PCR and Sanger sequencing were used to validate and genotype the *CACNA2D2* missense variant in two cases using the following primers: 5′‐TCAAGACAGATGGCCTCGTT‐3′ (forward primer) and 5′‐TGTTTGGGGCTTGAGGTTAC‐3′ (reverse primer). Additionally, 140 Swiss Angus controls were genotyped. PCR products from genomic DNA were amplified using AmpliTaq Gold 360 Master Mix (Thermo Fisher Scientific, Waltham, MA, USA) and the PCR amplicons were directly sequenced on an ABI3730 capillary sequencer (Thermo Fisher Scientific, Darmstadt, Germany).

Furthermore, the identified protein‐changing variant in *CACNA2D2* was added to the subsequently updated versions of the Swiss Axiom custom genotyping arrays (Thermo Fisher Scientific) routinely used for genomic selection in Switzerland. Thus, after conducting population‐wide genotyping of Swiss Angus cattle for genomic selection purposes, 194 genotypes for the variant were available. Subsequently, the prevalence of the identified *CACNA2D2* allele was evaluated.

## 3 Results

### 3.1 Clinicopathological Findings, Exclusion of Prevalent Teratogenic Virus and Pedigree Indicate a Genetic Form of Cerebellar Abiotrophy

On clinical examination, both calves were bright and alert but in permanent recumbency. Vital parameters revealed normothermia, tachycardia, and tachypnea. The ocular mucous membranes were diffusely congested.

At rest, both animals adopted sternal recumbency with intermittent nodding‐type head tremors and extension of the thoracic and pelvic limbs. Frequent spontaneous progression to lateral recumbency occurred, occasionally accompanied by opisthotonus. Passive manipulation of the limbs produced no resistance, and limb tone was within low‐normal limits. Stimulation to rise resulted in immediate collapse into lateral recumbency with marked exacerbation of the head tremors, preventing initiation of a quadrupedal stance.

Case 1, if passively positioned into a standing position, was able to transiently maintain a quadrupedal stance. Pelvic‐limb placement was appropriate; however, the thoracic limbs remained in dorso‐flexion of the fetlock joints (Video S1). Head tremors increased during attempted voluntary movement, compatible with intention tremors. Within 10–15 s, the calf developed carpal flexion, dorsocaudal extension of the neck and was only able to maintain partial weight‐bearing on the pelvic limbs with external support (Video S1). Unsupported stance could not be sustained. Progressive tremors and impaired postural reactions resulted in collapse into lateral recumbency. Tremor intensity decreased when the calf was unstimulated, allowing return to sternal recumbency.

Case 2 was unable to achieve a quadrupedal stance, even with full manual support. Both thoracic and pelvic limbs remained in persistent dorso‐flexion at the fetlock joints, and dorsocaudal extension of the head and neck occurred immediately, resulting in lateral recumbency. As in case 1, clinical signs were consistent with severe proprioceptive dysfunction, characterized by absent voluntary limb placement, impaired postural stability, and intention tremors.

Neurological examination revealed no abnormalities in cranial nerve function, segmental spinal reflexes, or nociceptive responses in either calf.

The combination of intention tremors, postural instability, and proprioceptive deficits was consistent with a cerebellar disorder.

Macroscopically, both calves displayed mild cerebral congestion, while the cerebellum appeared normal in size. Case 1 exhibited slight dilation of the lateral ventricles, whereas case 2 showed an increased volume of cerebrospinal fluid within the cranial cavity.

**FIGURE 1:**
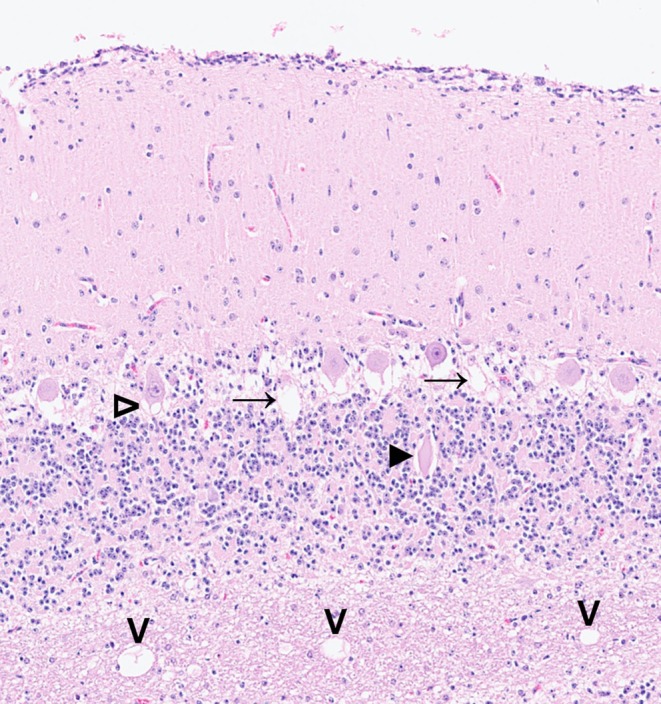
Histological findings of the cerebellum from an Angus calf affected by cerebellar abiotrophy. Purkinje cell cytoplasmic vacuolation (unfilled arrowhead) and axonal spheroid (filled arrowhead). Vacuolation of the basal molecular layer (arrows). White matter vacuoles (open arrowheads) occasionally contain cellular debris (left and middle open arrowheads). Case 1. Hematoxylin and eosin.

Histologically, in both cases, severe cerebellar cortical degeneration was observed (Figure 1), characterized by multifocal vacuolation of the basal molecular layer associated with alterations of the Purkinje neuronal dendritic tree, including occasional dendritic swelling. Purkinje neurons exhibited cytoplasmic vacuolation, chromatolysis, and occasional necrosis. Purkinje axonal spheroids (torpedoes) were noted. The internal granule layer was narrowed, with a reduced number of granule neurons. Variable vacuolation and occasional myelinoclastic vacuoles were present within the cerebellar white matter. Overall, these findings indicated pronounced Purkinje cell pathology and internal granule layer depletion consistent with severe cerebellar cortical degeneration.

Collectively, the clinical and histopathological findings were consistent with a congenital form of CA.

Tissue samples from both affected calves tested negative for BVDV, SBV, and BTV using PCR, excluding intrauterine viral infections with these currently prevalent agents as the cause of the observed phenotype.

Pedigree analysis indicated that the dam of case 1 was a granddaughter of the herd sire. In contrast, the dam of Case 2 was not closely related to the calf's sire. Considering the clinical and histopathological findings, the absence of evidence for teratogenic viral infections, and the pedigree information, a recessive inheritance pattern was suspected, prompting further genetic analysis.

### 3.2 Homozygosity Analyses

Regions of homozygosity (ROH) analyses were performed using the WGS. Genomic inbreeding coefficient (F_ROH) was estimated based on the detected ROH (Table S1). F_ROH of the affected calves was 0.41 (case 1) and 0.20 (case 2), which was considerably higher compared to the mean of F_ROH in the studied controls (mean 0.19, SD ± 0.03, range 0.13–0.23; Table S1), confirming the reported inbreeding, especially for case 1. The two affected Angus calves shared 534 ROH, 36 of which exceeded 1 Mb size (Figure 1a, Table S2). The three largest shared ROH were located on chromosomes 7 (~12.6 Mb, 7:68,796,855‐81,370,987), 15 (~12.3 Mb, 15:16,417,428‐28,694,737), and 22 (~6.3 Mb, 22: 46,586,946‐52,888,785). This analysis enabled reducing the potentially disease‐associated genomic regions to 3.8% of the bovine genome.

### 3.3 Identification of a Pathogenic Missense Variant in CACNA2D2

**FIGURE 2:**
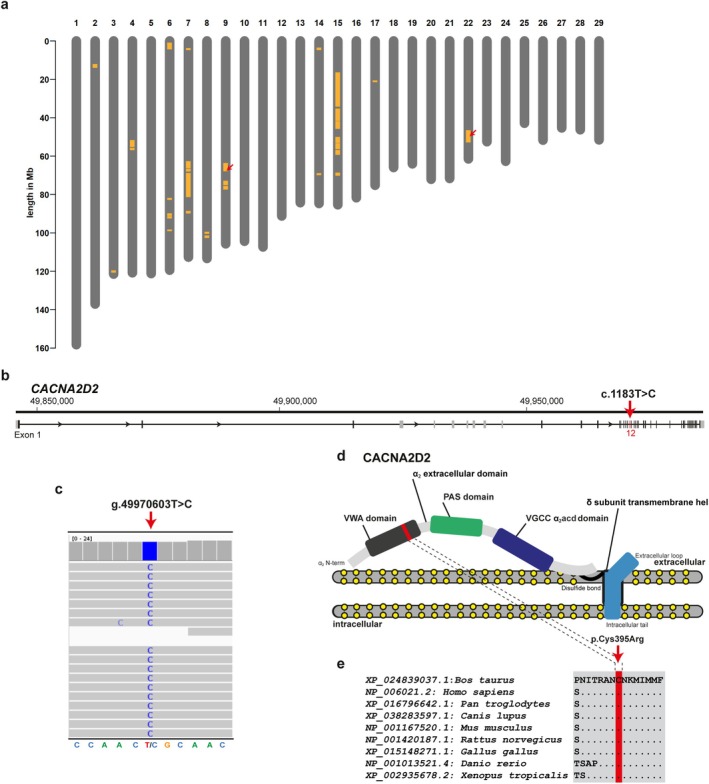
Identification of a cerebellar abiotrophy‐associated variant in two Angus calves. (a) Plot of runs of homozygosity (ROH) shared by the two cases, only the 36 ROH larger than 1 MB are shown (yellow segments) along 11 bovine chromosomes. The red arrows indicate location of the two protein‐changing SNVs absent from control genomes in a homozygous state. (b) *CACNA2D2* gene structure showing the location of the candidate causal variant XM_024983269.1: c.1183T>C on chromosome 22, exon 12 (red arrow). (c) IGV screenshot of the genome of an affected calf showing the homozygous missense variant chr22: g.49970603T>C. (d) Schematic representation of the bovine CACNA2D2 protein. The position of the XP_024839037.1: p.(Cys395Arg) variant is indicated by the red arrow. (e) Multiple sequence alignment of the CACNA2D2 protein encompassing the region of the affected residue reveals complete evolutionary conservation across species.

Subsequently, the established SNV data were filtered for candidate causal variants. A total of 17 protein‐changing sequence variants were identified for which the two affected calves were homozygous for the variant allele and 1033 controls were heterozygous or homozygous for the reference allele (Table S3). Occurrence of these variants was further investigated using the 1000 Bull Genomes Project. Fifteen of these variants appeared in the homozygous variant status in this global cohort. From the two remaining SNVs, only one affected a plausible candidate gene for neurodevelopmental disorders (Table S3). This was a homozygous SNV in *CACNA2D2* exon 12 (chr22:g. 49 970 603T>C; XM_024983269.1: c.1183T>C), which was confirmed by visual inspection and subsequent Sanger sequencing (Figure 2b,c). The *CACNA2D2* missense variant was predicted to change the encoded amino acid of CACNA2D2 residue 395 (XP_024839037.1: p.Cys395Arg), which is located in the VWFA domain (Figure 2d). In addition, the variant was predicted to be deleterious by all the used in silico tools (Table S4) and the affected amino acid was highly conserved across species revealing complete evolutionary conservation (Figure 2e). In contrast, the second missense variant affecting the *LAMA2* gene was predicted to be neutral and occurred heterozygous in 22 cattle from the global cohort in Angus, Red Danish, and Brown Swiss (Table S4). The *CACNA2D2* variant allele was not detected in any of the control animals, suggesting that it is either extremely rare or absent from the broader global population. Furthermore, the variant frequency was investigated in the genotyped 334 Swiss Angus cattle and was also found to be absent.

## 4 Discussion

We describe the clinical and pathological features of two paternal half‐sib Angus calves affected by a *CACNA2D2*‐related form of CA. Clinically, the calves exhibited intention tremors, postural instability, and proprioceptive deficits consistent with a primary cerebellar disorder. Histopathological examination confirmed the clinical suspicion by demonstrating marked degeneration and loss of Purkinje cells, consistent with CA. Histopathological examination confirmed the clinical suspicion, revealing marked degeneration and loss of Purkinje cells, findings characteristic of CA. Genetic analysis identified a homozygous missense variant in *CACNA2D2*, which was considered pathogenic based on its rarity, co‐segregation with the disease in affected individuals, in silico predictions of functional impact, and conservation of the affected amino acid (Boeykens et al. 2024). Taken together, the clinical, histopathological, and genetic evidence supports the *CACNA2D2* variant as the cause of the observed congenital CA in the studied Angus calves.

*CACNA2D2* encodes the voltage‐dependent calcium channel subunit alpha‐2/delta‐2 protein, which plays a critical role in regulating calcium influx into neurons (Edvardson et al. 2013; Lacinová and Klugbauer 2004). It acts as a regulatory subunit for multiple calcium channel types, including P/Q‐type (*CACNA1A*), N‐type (*CACNA1B*), L‐type (*CACNA1C*, *CACNA1D*), and potentially T‐type (*CACNA1G*) channels (Edvardson et al. 2013; Lacinová and Klugbauer 2004). Interestingly, a different bovine familial neurodevelopmental disorder affecting the same breed of cattle, clinically characterized by convulsions and ataxia, observed in the progeny of a single Angus sire, could be explained by a spontaneous dominant de novo mutation in *CACNA1A*, representing a germline mosaic (Reith et al. 2024).

Spontaneous disease‐associated *CACNA2D2* variants have been reported in both humans and mice (Barclay et al. 2001; Edvardson et al. 2013). In humans, pathogenic variants in the *CACNA2D2* are associated with cerebellar atrophy with seizures and variable developmental delay (OMIM 607082), which is a rare, early‐onset, recessively inherited disorder. It is characterized by refractory seizures, global developmental delay, cerebellar ataxia and vermis atrophy (Butler et al. 2018; Edvardson et al. 2013; Pippucci et al. 2013; Valence et al. 2019). While most patients exhibit severe symptoms such as axial hypotonia, abnormal eye movements and inability to walk, milder phenotypes with preserved cognitive function have also been documented, implying clinical heterogeneity (Valence et al. 2019).

Homozygous *Cacna2d2* ^du‐2J^ mutant mice (MGI:2149331), carrying a spontaneous 2 bp frameshift deletion in exon 9, exhibit cerebellar ataxia, abnormal Purkinje cell morphology and size, impaired synaptic transmission, and absence seizures, despite stable mRNA levels, indicating that the truncated protein disrupts normal cerebellar and neuronal function (Barclay et al. 2001). Additionally, homozygous *Cacna2d2* ^du^ mutant mice (MGI:1856022) harboring a spontaneous complex genomic rearrangement causing loss of function display a severe neurological phenotype including ataxia, abnormal gait, seizures, hindlimb paralysis, and stationary movements (Barclay et al. 2001; Brodbeck et al. 2002; Holz et al. 1997). Neuropathologically, these mice show marked reduction in Purkinje cell number and degeneration, abnormal neuron and glial cell morphology, decreased brain and spinal cord size, and defects in hindbrain structures such as the medulla and pons (Barclay et al. 2001; Brodbeck et al. 2002; Walter et al. 2006). They also exhibit abnormalities in synaptic transmission, axonal dystrophy, and impaired myelination, leading to progressive neurodegeneration and premature death (Barclay et al. 2001; Brodbeck et al. 2002; Walter et al. 2006).

Altogether, the known genotype–phenotype correlations emphasize the critical role of *CACNA2D2* in cerebellar development, motor coordination, and neuronal and synaptic integrity. These features align closely with the clinical and pathological findings in the CA‐affected Angus calves, which clinically showed severe tremors, permanent recumbency, and histopathological evidence of cerebellar abiotrophy. The prominent cerebellar lesions in the CA‐affected calves are speculated to be consistent with disrupted calcium homeostasis and impaired maturation of cerebellar circuits during neurodevelopment.

Analysis of genotyping data from a large global cattle cohort, including animals from the 1000 Bull Genomes Project, and a small group of Angus cattle in Switzerland, revealed no evidence of the variant allele. This suggests that the identified variant in *CACNA2D2* is either extremely rare or entirely absent from the broader global cattle population, as well as from the Swiss Angus cattle cohort studied. This indicates that the variant may also be rare or absent outside the cases identified in the United Kingdom. This supports the hypothesis of a recessively inherited disorder with a local founder effect, underlining the importance of population‐specific genetic surveillance to manage and prevent the spread of deleterious alleles within breeding programs (Charlier et al. 2008; Daetwyler et al. 2014). Together, these data imply that the identified *CACNA2D2* variant may represent a rare deleterious allele private to an isolated subpopulation, arising due to a recent mutation event considering the linked ROH size of ~ 6.3 Mb. This emphasizes the need for targeted genetic testing and breeding strategies in affected regions to prevent the propagation of the pathogenic allele.

A limitation of this study is the lack of direct functional assays confirming the impact of the identified *CACNA2D2* variant in the encoded protein. Larger screening of *CACNA2D2* variants in bigger and more diverse cohorts, in particular in the affected purebred British Angus population, is warranted in future studies.

## 5 Conclusion

For the first time, we characterized a genetic form of cerebellar disease in cattle, providing the first large animal model for a condition related to the *CACNA2D2* gene. Our findings expand the known spectrum of *CACNA2D2*‐related neurodevelopmental disorders across species. The apparent geographic confinement of this allele points to a recent origin and local founder effect, underscoring the need for targeted genetic screening programs to prevent further dissemination of such deleterious alleles within the breeding population.

## Author Contributions

**Joana Jacinto:** conceptualization, formal analysis, visualization, writing – original draft, investigation, writing – review and editing, validation, data curation. **Francesca Chianini:** conceptualization, methodology, investigation, data curation, formal analysis, writing – review and editing. **Jo Moore:** conceptualization, data curation, methodology, investigation, writing – review and editing, formal analysis. **Timothy Geraghty:** conceptualization, investigation, methodology. **Irene M. Häfliger:** formal analysis, visualization, software, methodology. **Franz R. Seefried:** formal analysis, visualization, methodology, software, writing – review and editing. **Alwyn Jones:** formal analysis, methodology, investigation. **Helen Carty:** data curation, formal analysis, methodology, investigation, writing – review and editing. **Anna Letko:** data curation, visualization, formal analysis, writing – review and editing, methodology, software, validation. **Cord Drögemüller:** conceptualization, writing – review and editing, supervision, project administration, resources, visualization.

## Funding

Joana Jacinto is supported in part by the Arbeitsgemeinschaft Schweizerischer Rinderzüchter (ASR), the Swiss Federal Office for Agriculture (BLW), and the Faculty Clinical Research Platform (FCRP) of the Vetsuisse Faculty of the University of Bern.

## Conflicts of Interest

The authors declare no conflicts of interest.

## Supporting information


**Table S1:** Total size of individual ROH and genomic inbreeding coefficient in all 18 Angus cattle genomes.

**Table S2:** Shared ROH detected in the two Angus cases.

**Table S3:** Detailed description of the homozygous protein changing variants shared by two CA‐affected Angus calves and their frequencies in the global control cohort.

**Table S4:** Detailed pathogenicity classification of the *CACNA2D2* and *LAMA2* variants.

**Video S1:** Clinical phenotype of the CA‐affected Angus calves (case 1 and 2).

## Data Availability

The WGS data are available under the Swiss Comparative Bovine Resequencing Project study accession no. PRJEB18113 and PRJEB83441 at the European Nucleotide Archive (www.ebi.ac.uk/ena; case 1: SAMEA12596611; case 2: SAMEA12596612).
